# Face mask sampling (FMS) for tuberculosis shows lower diagnostic sensitivity than sputum sampling in Guinea

**DOI:** 10.1186/s12941-023-00633-8

**Published:** 2023-09-07

**Authors:** Souleymane Hassane-Harouna, Sofie Marijke Braet, Tom Decroo, Lansana Mady Camara, Alexandre Delamou, Sven de Bock, Nimer Ortuño-Gutiérrez, Gba-Foromo Cherif, Caroline M. Williams, Anika Wisniewska, Michael R. Barer, Leen Rigouts, Bouke Catherine de Jong

**Affiliations:** 1 Action Damien Conakry, Conakry, Guinea; 2 Research Foundation Flanders, Brussels, Belgium; 3 Unit of Mycobacteriology, Department of Biomedical Sciences, Institute of Tropical Medicine, Antwerp, Belgium; 4 Unit of HIV & Co-infections, Department of Clinical Sciences, Institute of Tropical Medicine, Antwerp, Belgium; 5 Gamal Abdel Nasser University, Conakry, Guinea; 6 Damien Foundation, Brussels, Belgium; 7 Department of Infection, Immunity and Inflammation, University of Leicester, Leicester, UK; 8 Department of Clinical Microbiology, University Hospitals of Leicester NHS Trust, Leicester, UK

**Keywords:** Face mask sampling, Pulmonary tuberculosis, Guinea

## Abstract

**Background:**

Pulmonary tuberculosis (PTB) diagnosis relies on sputum examination, a challenge in sputum-scarce patients. Alternative non-invasive sampling methods such as face mask sampling (FMS) have been proposed.

**Objective:**

To evaluate the value of FMS for PTB diagnosis by assessing its agreement with sputum samples processed by GeneXpert MTB/RIF (Ultra)(Xpert) testing, and describe FMS sensitivity and specificity.

**Methods:**

This was a prospective study conducted at the Carrière TB clinic in Guinea. Presumptive TB patients willing to participate were asked to wear a surgical mask containing a polyvinyl alcohol (PVA) strip for thirty minutes. Subsequently, two spot sputum samples were collected, of which one was processed by microscopy on site and the other by Xpert in Guinea’s National Reference Laboratory of Mycobacteriology (LNRM). The first 30 FMS were processed at the Supranational Reference Laboratory in Antwerp, Belgium, and the following 118 FMS in the LNRM.

**Results:**

One hundred fifty patients participated, of whom 148 had valid results for both mask and sputum. Sputum smear microscopy was positive for 47 (31.8%) patients while sputum-Xpert detected MTB in 54 (36.5%) patients. Among the 54 patients testing sputum-Xpert positive, 26 (48.1%) yielded a positive FMS-Xpert result, while four sputum-Xpert negative patients tested positive for FMS and 90 patients were Xpert-negative for both sputum and mask samples, suggesting a moderate level of agreement (k-value of 0.47). The overall mask sensitivity was 48.1%, with 95.7% specificity.

**Conclusion:**

In our setting, Xpert testing on FMS did not yield a high level of agreement to sputum sample.

**Supplementary Information:**

The online version contains supplementary material available at 10.1186/s12941-023-00633-8.

## Introduction

Tuberculosis (TB) remains the leading cause of death from a single infectious disease except during the year 2020. The World Health Organization (WHO) 2022 report estimated that 10.6 million people developed TB disease in 2021. However, only 6.4 million patients were diagnosed and reported in 2021 representing a detection gap of over 4 million patients. Of those diagnosed, only 3.4 million (53%) had bacteriologically confirmed pulmonary TB [1].

Fast, sensitive and accessible diagnostics are required to reduce the diagnostic gap and increase treatment coverage. Pulmonary TB diagnosis is based on clinical presentation, chest X-ray investigation and results from diagnostic tests (smear microscopy, rapid molecular tests, and culture), usually done on sputum samples [2, 3]. However, sputum production can be challenging for specific groups, such as people living with HIV and children, as well as the elderly and patients without productive cough [3–5].

From 2014, a novel strategy was evaluated, using face masks adapted with a gelatine filter to capture airborne bacilli [6]. Early results showed that 65% of patients with confirmed sputum smear-positive pulmonary TB had a positive result from mask samples analysed by Xpert. In six patients diagnosed by broncho-alveolar lavage (BAL), 3 were mask positive and for one of these, the BAL was negative on smear and culture. When clinical diagnosis of PTB was used for comparison, none of these patients tested false positive [6]. Preliminary data on the use of FMS containing two 3D-printed polyvinyl alcohol (PVA) strips, instead of gelatine filters, were obtained in Pretoria (South Africa) [7]. Both sputa and mask samples were analysed from 20 presumptive TB patients. Eight were diagnosed with PTB and six out of them were exclusively MTB positive with mask-Xpert [7]. Follow-up of these mask-positive/sputum-negative patients confirmed active PTB disease (sputum-Xpert positive) in four patients six weeks later, while one still remaining sputum-Xpert negative at 20 weeks had completed a TB treatment of 6 months prior to be included in the study. The sixth patient was lost to follow up. Thus, even though aerosol sampling by masks may not replace sputum sampling, this data suggest that masks may increase the laboratory confirmation of (clinically) diagnosed TB, especially if substantiated by clinical presentation to confirm active TB disease. Especially in household contacts of TB patients, who often present at an early (pre-symptomatic) stage and with paucibacillary TB [8], FMS for Xpert testing may diagnose TB when the sputum-Xpert is negative or not possible (e.g. “dry” cough).

The aim of this study was to evaluate the value of FMS for PTB diagnosis by assessing its agreement with sputum-Xpert testing and describe its sensitivity and specificity compared to sputum sampling.

## Methodology

### Study design, setting and population

This prospective TB diagnostic study was conducted at the Centre Antituberculeux de Référence la Carrière (CATR), the most frequented TB clinic of Guinea, a country with a population of 14 million. TB incidence is estimated at 175 per 100,000 persons, with 29% of these estimated TB patients not being diagnosed [9]. In Guinea, the prevalence of HIV among TB patients is 22% [10].

Presumptive TB patients aged 15 years old or more attending the CATR from April 2019 to December 2020 were invited for this study. Patients not willing to provide consent or not able to wear a mask for thirty minutes (including patients with a debilitated physical condition) were excluded.

### Sample size

Studies on active case finding show that TB can be diagnosed in 40% or more among those with presumptive TB and tested with Xpert MTB/RIF [11, 12]. Assuming that 40% of patients with presumptive TB will have either a positive mask or sputum result, and assuming that 10% of patients with presumptive TB will be mask-positive/sputum-negative (in 139 patients 10% can be estimated with 5% precision and 95% confidence), and that we have a few invalid/ error results and few patients lost-to follow up along the diagnostic pathway (not more than 7% loss), the enrolment of 150 adults with presumptive TB was envisaged.

### Study procedures

#### Mask description and sampling procedure

We used duckbill surgical masks containing two PVA strips [7]. Each mask was packed in a closed plastic bag accompanied by a 4 ml spray bottle containing molecular grade water, and stored at ambient temperature in a cabinet located in the consultation room.

To sample a patient, one mask was removed from the plastic bag. Then the membrane was pre-moistened by spraying with the molecular grade water. Trained study staff (physicians and nurses) supervised the sampling process and adjusted the mask position if needed. They instructed the patient to keep it on for thirty minutes during which (s)he could breath, talk, cough, or sneeze as usual. Sampling was done in open air at the health facility. The masks were removed from the patients by the supervisor and packed in the original plastic bag. While wearing the mask, the patients held a spit pot in which they could spit at any time. Subsequently, they were requested to provide a (second) sputum shortly after the mask sample collection, to have a total of two spot sputum specimens with minimum 5 ml each per patient.

### Sputum samples processing

Sputum samples were processed with Xpert testing as per manufacturer’s protocol. One sample of each patient was directly processed by microscopy at the CATR laboratory while the other one was processed by Xpert at the National Reference Laboratory of Mycobacteriology (LNRM) of Conakry.

### Mask samples processing

Due to shortage of technical material, the first 30 FMS could not be processed at LNRM as planned. They were stored at 2–8 °C at the LNRM until a shipment to the Institute of Tropical Medicine (ITM) in Antwerp could be arranged. The median (IQR) duration (in days) between sampling and processing at ITM was 62.5 (57–70). To process the masks at ITM, the strips were first removed from the masks by cutting with scissors; the scissors were cleaned with alcohol and autoclaved between use. Strips were then transferred in plastic bags into which 5 mL molecular-grade water was added and the mixture dissolved manually. The mixture was finally transferred into a sterile 15 mL Falcon tube and 140 µL Xpert sample reagent was added and mixed well. Finally, 2mL of the mixture was loaded into an Xpert MTB/RIF cassette and loaded into the GeneXpert machine.

The remaining 120 FMS were processed at the LNRM of Conakry, as foreseen. The main point to emphasize here is that the strips were dissolved by an automate. After removal from the masks, the strips were transferred into a sterile 15 mL Falcon tube containing 5 mL molecular-grade water. Subsequently, tubes were loaded onto a shaker (Multi-rotator PTR-35 GRANT-bio, Version V.5GW, Grant Instruments LTD, England) to dissolve the strips. Apart from the manual strip dissolution for the first 30 masks and the automated system used for the last 120 masks, the remaining processing steps were the same for all masks. In Conakry, both GeneXpert MTB/RIF and GeneXpert MTB/RIF Ultra cartridges were used, depending on availability. Paired mask and sputum specimens were always processed in the same type of Xpert cartridge within one day of collection.

Sputum-Xpert results were considered as the reference.

### Patient management and follow up

Patients with a positive sputum-Xpert result received appropriate anti-TB treatment with monthly follow up, as per national guidelines. As mask sampling is not endorsed by the national TB programme, patients with a mask+/sputum- result could not be started on TB treatment. They were treated with broad-spectrum antibiotics, with monthly follow-up for one year to assess for TB and initiate anti-TB treatment in case sputum examination and/or clinical signs would support TB diagnosis. Patients with a sputum-/mask- result were considered as not TB patients and were not subject to any particular follow-up within the framework of the study.

### Statistical analysis

All analyses were conducted with R version 4.1.1 for Windows (The R foundation, Vienna, Austria). To determine the level of agreement between the mask and sputum sample results, the *vdr* package was used to calculate the kappa value.

## Results

From April 2019 to December 2020, a total of 159 presumptive TB patients were offered mask sampling, of whom nine declined participation and were therefore not included in the study. In total, 150 patients participated in the study, of which 148 had either “MTB detected” or “MTB not detected” for both the mask and the sputum sample. Two patients with “Invalid” Xpert-mask results were excluded from the final analysis; one had “MTB detected” and the other “MTB not detected” by Xpert testing from sputum. No “Invalid” results were obtained for sputum-based Xpert testing.

Of 148 patients with presumed PTB, 135 (91.2%) were new presumptive TB patients and 13 (8.8%) had received previous anti-TB treatment. Additionally, 10 (7.4%) of these new presumptive TB patients were known contacts of individuals who had confirmed TB. HIV was positive in 16 (11.1%) of 143 tested patients. Sputum smear microscopy identified acid-fast bacilli in 47 (31.8%) patients (Table 1).

Xpert on sputum was positive in 54 (36.5%) patients, of whom Xpert on mask was positive in 26 (17.6%, Table 1).

**Table 1:** Mask-Xpert and sputum-Xpert results, by patient and diagnostic characteristics

		Sputum positive *n = 54 (36.5)*		Sputum negative *n = 94 (63.5)*	
		**Mask positive**	**Mask negative**	**Mask positive**	**Mask** **negative**
	**N (%)**	N (%)	N (%)	N (%)	N (%)
Total	**148 (100.0)**	26 (48.1)	28 (51.9)	4 (4.3)	90 (95.7)
HIV status					
Tested	**143 (96.6)**	26 (100.0)	26 (92.9)	4 (100.0)	87 (96.7)
Positive	**16 (11.1)**	6 (23.0)	4 (15.3	1 (25.0)	5 (5.7)
TB Type					
New presumptive TB patient	135 (91.2)	25 (96.1)	26 (92.9)	4 (100.0)	80 (88.9)
Prev. Tr*	13 (8.8)	1 (3.9)	2 (7.1)	0 (0.0)	10 (11.1)
TB Cont**					
Yes	10 (6.8)	0 (0.0)	2 (7.1)	0 (0.0)	8 (8.9)
No	138 (93.2)	26 (100.0)	26 (92.9)	4 (100.0)	82 (91.1)
SSM					
Negative	101 (68.2)	1 (3.8)	6 (21.4)	4 (100.0)	90 (100.0)
Positive	47 (31.8)	26 (100.0)	22 (78.6)	0 (0.0)	0 (0.0)
Xpert					
Classic	78 (52.7)	9 (34.6)	18 (64.3)	0 (0.0)	51 (56.7)
Ultra	70 (47.3)	17 (65.4)	10 (35.7)	4 (100.0)	39 (43.3)

*Previously treated, ** contact of known tuberculosis patient; N = number of samples; SSM = sputum smear microscopy; TB = tuberculosis

In four patients, mask results were positive while sputum results were negative. This corresponded to 95% observed agreement and a k value of 0.47, suggesting a moderate level of agreement.

The overall mask sensitivity compared to sputum-Xpert testing was 48.1%, with 95.7% specificity. Xpert sputum result was considered the gold standard for sensitivity calculation, (Table 2).

Considering only the 118 patients for whom samples were processed locally at the LNRM, 48 samples were sputum-Xpert positive of which 29 were positive on mask, showing a sensitivity of 52.1% with 94.2% specificity. Of the 30 patients for whom masks samples were processed at ITM, six were sputum-Xpert in Guinea, of which only 1 had a positive FMS-Xpert result, yielding 16.7% sensitivity only (Table 2).

**Table 2:** Face mask samples sensitivity and specificity relative to sputum-Xpert results

	ITM (N = 30)		LNRM (N = 118)		ITM + LNRM (N = 148)	
	Sput-Xpert+	Sput-Xpert-	Sput-Xpert+	Sput-Xpert-	Sput-Xpert+	Sput-Xpert-
Mask-Xpert+	1	0	25	4	26	4
Mask-Xpert-	5	24	23	66	28	90
Sen (%)	***16.7***		***52.1***		***48.1***	
Spec (%)	100		**94.2**		**95.7**	

Sput = sputum; + = positive; - = negative; N = number of samples; Sen = sensitivity; Spec = specificity; ITM = Institute of Tropical Medicine, Antwerp, Belgium; LNRM = Laboratoire National de Référence de Mycobactériologie, Conakry, Guinée.

Stratification of FMS-Xpert results by type of Xpert cartridge, showed a relative higher sensitivity for Xpert Ultra compared to Xpert MTB/RIF. Of the 118 paired samples processed at the LNRM, 70 were performed with Xpert Ultra and 48 with Xpert MTB/RIF. Of the 27 sputum-positive on Xpert Ultra, 17 were mask-positive, while four FMS were positive with a negative sputum-Xpert result. Hence, sensitivity for Xpert Ultra mask testing was 62.9% with 90.6% specificity (Table 3a).

**Table 3a:** Face mask samples sensitivity and specificity using Xpert Ultra testing

LNRM (n = 70)	Nb sput+	Nb sput-
**Nb mask+**	**17**	4
**Nb mask-**	**10**	**39**
**Sensitivity: 62.9% Specificity: 90.6%**		

For the 48 patients tested by Xpert MTB/RIF, 21 samples were sputum-Xpert positive of which eight were mask-Xpert positive, showing a lower mask-Xpert sensitivity of 38.0% compared with 100% specificity (Table 3b).

**Table 3b:** Face mask samples sensitivity and specificity using Xpert MTB/RIF

LNRM (n = 48)	Nb sput+	Nb sput-
Nb mask+	8	0
Nb mask-	13	27
**Sensitivity: 38.0% Specificity: 100.0%**		

Regarding the detected bacillary load, except for one patient (N°147), all patients with positive Xpert results for both sputum and FMS were found to be positive on smear microscopy. Likewise, except for one patient (N° 73), the bacillary load detected with Xpert in sputum-positive samples was consistently higher than the bacillary load in the respective mask-positive samples, irrespective of the Xpert cartridge type used (Table 4, please consider as supplementary table).

As per protocol and ethical approval, only patients who tested sputum positive were initiated on TB treatment. After one year follow-up of the four patients who tested positive only on FMS but negative on sputum, the one with HIV coinfection was clinically diagnosed with lymph node TB two months after FMS positivity, two improved clinically after a two-week amoxicillin-clavulanic acid treatment and were declared to not have TB disease, while the fourth was lost to follow-up.

Regarding the patient’s history, only 13 (8.8%) patients experienced a previous TB episode, of which 3 were diagnosed sputum-positive by Xpert and initiated on TB treatment. Only one of them was mask-positive on Xpert. No mask-positive/sputum-negative previously treated TB patients were identified.

Among the 16 HIV-coinfected patients, ten yielded a sputum-positive Xpert result and seven a mask-positive Xpert result. Only one had a mask-positive/sputum-negative result.

## Discussion

Our findings confirm that FMS is able to detect *Mycobacterium tuberculosis* (MTB) from presumed PTB patients through aerosols. While the FMS detection rate was lower than sputum, this difference was smaller when using Xpert Ultra compared to the classical Xpert MTB/RIF. In Xpert Ultra, FMS identified MTB in four additional patients compared to sputum, all with a ‘trace’ positive result, of whom only one of three who could be ascertained during one year follow-up was confirmed to develop TB lymphadenitis soon (2 months) after the positive FMS result.

Limiting to the freshly processed samples in Guinea, mask and sputum samples MTB detection rates were respectively 24.6% and 40.3% with a consistent lower bacillary load detected by mask sampling. Our findings show a lower overall sensitivity than previously observed in Pretoria, the Gambia/UK, despite applying the same type of masks and processing procedure in Guinea as in Pretoria [7]. The reasons for the lower sensitivity obtained in Guinea remain unclear. HIV co-infection rates were higher in the Pretoria study (20% of presumed TB patients with HIV coinfection) compared to our study population (11.3%). The sputum-based bacillary load in the Pretoria study was lower than in our study. Xpert Ultra was used for sample processing in the Pretoria study while in Gambia/UK study, the classical Xpert MTB/RIF was used. In our study, samples were processed at ITM by Xpert MTB/RIF while in Guinea, both types of cartridges were used according to their availability, with preferred used of Xpert Ultra when the two types were available.

When only considering Xpert Ultra results from our study, the MTB detection rate increased to 30.0% for masks while it slightly decreased for sputum (38.6%), as compared to classical Xpert MTB/RIF testing. The same trend of higher sensitivity for Xpert Ultra was observed for mask sensitivity relative to sputum. These findings suggest that an improvement of mask sensitivity is possible when using Xpert Ultra. Also testing on fresh specimens may improve sensitivity, as suggested by the decreased positivity rate following storage and remote testing at ITM in our study.

Mask sampling offers additional opportunities beyond the diagnostic advantage in persons unable to cough up sputum. Indeed, FMS testing also informs the natural history of TB and its transmissibility. Williams and colleagues demonstrated in their recent study that FMS enables the stratification of patients with high risk of infectiousness, especially in settings with a high TB-burden [13]. Importantly, the greatest proportion of MTB was shown to be exhaled during normal ‘tidal’ breathing, even if more bacilli were expelled during a cough. Cough frequency correlated poorly with the number of exhaled bacilli [14, 15]. Indeed, the reservoir of undiagnosed TB is likely much larger than the estimated “missing three millions” in the WHO report, when taking into account evidence from prevalence surveys showing that less than half of patients with microbiologically confirmed TB report symptoms [16]. A proportion of these patients may be detectable by mask only.

FMS based Xpert testing has a high pooled specificity, both for Xpert MTB/RIF (100%, Table 3b) and Xpert Ultra (90.6%, Table 3a), even though the specificity of Xpert Ultra trace results in previously treated patients is questioned. In our study, no trace result was observed among the previously treated patients, while the only four patients that yielded a mask-positive/sputum-negative result by Xpert Ultra testing, had a trace result. They were all new presumptive TB patients, of which one developed TB lymphadenitis two months after the positive FMS result. As no duplicate sputum or FMS testing was done at initial diagnosis and inter-specimen bacillary load may vary [17], we can’t conclude from this single case that FMS was more sensitive to detect TB in this patient at the early stage. Two of the patients were probable false-FMS results as they cleared symptoms after non-TB treatment and did not develop TB within a one-year follow-up period, while the fourth patient could not be ruled out. The reason for these false FSM trace results remains unresolved.

Finally, the automated strip dissolving was used to save time by processing multiple masks simultaneously. There is no evidence that this method results in a higher sensitivity for MTB detection compared to the manual method.

### Study limitations

Mask samples were not all processed in the same laboratory. Consequently, sample processing in Guinea was done within 48 h, while those processed at ITM were done up to two months after sampling. From the ITM-processed samples, many had the strips very stuck to the masks rendering their removal quite laborious. This might have led to DNA loss and/or degradation and could explain the observed difference in sensitivity between masks processed at ITM and those processed in Guinea.

The 120 patients for whom mask samples were processed in Guinea were recruited during the COVID-19 crisis. During this period, the attendance at all health facilities decreased drastically and only very symptomatic patients were willing to seek medical consultation. This may represent a bias in the selection of presumptive TB patients.

## Conclusion

In our setting FMS showed lower sensitivity for PTB diagnosis relative to sputum sampling, and little added value in Xpert Ultra only. However, we confirm the FMS’s ability to detect MTB and feasibility in programmatic conditions. This strategy may complement empirical sputum sampling especially in patients with difficulty to produce sputum. FIND (Geneva, Switzerland) now evaluates face masks with a larger surface to capture exhaled bacilli. Alternative sampling strategies could be considered too, as studies using tongue swab sampling to diagnose pulmonary TB have been reported with promising preliminary results [2, 10].

### Electronic supplementary material

Below is the link to the electronic supplementary material.

Supplementary Material 1

## Data Availability

The datasets used and analysed during the current study are available from the corresponding author on reasonable request.
