# Supplementary material for: Face mask sampling (FMS) for tuberculosis shows lower diagnostic sensitivity than sputum sampling in Guinea
Source: Ann Clin Microbiol Antimicrob. 2023 Sep 7;22:81. doi: 10.1186/s12941-023-00633-8 (PMC10486030; doi:10.1186/s12941-023-00633-8)
Supplement: Supplementary file 1 — Supplementary Material 1 [file 12941_2023_633_MOESM1_ESM.docx]

**Supplementary table**

**Table 4: Overview of 58 presumptive tuberculosis (TB) patients yielding a positive result by Xpert testing from sputum and/or mask samples, stratified per type of test used, lab that performed the analysis, and HIV status and TB history of the patient.**

| **Patient**  **No** | **HIV**  **status** | **TB**  **history** | **AFB**  **smear** | **Mask**  **Xpert** | **Sputum**  **Xpert** | **Xpert**  **type** | **Lab** |
| --- | --- | --- | --- | --- | --- | --- | --- |
| 014 | Negative | New patient | 1+ | Negative | Medium | Classic | ITM |
| 015 | Negative | New patient | 2+ | Negative | High | Classic | ITM |
| 016 | Negative | New patient | Scanty | Very low | Medium | Classic | ITM |
| 020 | Negative | New patient | 3+ | Negative | High | Classic | ITM |
| 024 | Negative | New patient | 2+ | Negative | Medium | Classic | ITM |
| 027 | Negative | Previous TB | 3+ | Negative | Low | Classic | ITM |
| 031 | Negative | New patient | 2+ | Very low | High | Ultra | LNRM |
| 041 | Negative | New patient | 2+ | Very low | Medium | Classic | LNRM |
| 045 | Negative | New patient | 1+ | Negative | Low | Classic | LNRM |
| 047 | Positive | New patient | 2+ | Negative | Medium | Classic | LNRM |
| 050 | Negative | New patient | 1+ | Negative | Medium | Classic | LNRM |
| 051 | Negative | New patient | 1+ | Negative | High | Classic | LNRM |
| 054 | Negative | New patient | 3+ | Negative | Medium | Classic | LNRM |
| 056 | Positive | New patient | Scanty | Negative | Very Low | Classic | LNRM |
| 061 | Negative | New patient | 2+ | Very low | Medium | Classic | LNRM |
| 064 | Negative | New patient | Negative | Negative | Very Low | Classic | LNRM |
| 065 | Positive | New patient | 2+ | Very low | Medium | Classic | LNRM |
| 066 | Negative | New patient | 2+ | Negative | Medium | Classic | LNRM |
| 067 | Negative | New patient | 1+ | Negative | Medium | Classic | LNRM |
| 068 | Negative | New patient | 2+ | Low | Medium | Classic | LNRM |
| 072 | Negative | Previous TB | 3+ | Low | High | Classic | LNRM |
| **073** | Negative | New patient | 2+ | Medium | Medium | Classic | LNRM |
| 075 | Negative | New patient | 2+ | Very low | Medium | Classic | LNRM |
| 077 | Negative | New patient | 2+ | Negative | Medium | Classic | LNRM |
| 078 | Negative | New patient | Negative | Negative | Very Low | Classic | LNRM |
| 079 | Negative | New patient | 2+ | Negative | Medium | Classic | LNRM |
| 080 | Negative | New patient | Negative | Negative | Very Low | Classic | LNRM |
| 089 | Negative | New patient | Scanty | Very low | Low | Classic | LNRM |
| 091 | Positive | New patient | 2+ | Trace | High | Ultra | LNRM |
| 094 | Negative | New patient | Scanty | Trace | Low | Ultra | LNRM |
| 095 | Negative | New patient | 3+ | Negative | High | Ultra | LNRM |
| 096 | Positive | New patient | Negative | Trace | Negative | Ultra | LNRM |
| 104 | Positive | New patient | 3+ | Very low | High | Ultra | LNRM |
| 105 | Negative | New patient | Scanty | Negative | Low | Ultra | LNRM |
| 106 | Negative | New patient | Negative | Trace | Negative | Ultra | LNRM |
| 107 | Negative | New patient | Negative | Negative | Low | Ultra | LNRM |
| 109 | Negative | New patient | 3+ | Negative | High | Ultra | LNRM |
| 111 | Negative | New patient | Negative | Trace | Negative | Ultra | LNRM |
| 112 | Negative | New patient | 3+ | Very low | High | Ultra | LNRM |
| 113 | Negative | New patient | 1+ | Low | Low | Ultra | LNRM |
| 114 | Negative | New patient | 3+ | Very low | High | Ultra | LNRM |
| 115 | Negative | New patient | 1+ | Low | Medium | Ultra | LNRM |
| 116 | Negative | New patient | 3+ | Trace | High | Ultra | LNRM |
| 117 | Negative | New patient | Negative | Trace | Negative | Ultra | LNRM |
| 120 | Positive | New patient | 2+ | Very low | Medium | Ultra | LNRM |
| 123 | Negative | New patient | 2+ | Trace | High | Ultra | LNRM |
| 124 | Positive | Previous TB | Negative | Negative | Very Low | Ultra | LNRM |
| 126 | Negative | New patient | Scanty | Negative | Low | Ultra | LNRM |
| 128 | Positive | New patient | 2+ | Negative | Medium | Ultra | LNRM |
| 129 | Negative | New patient | Negative | Negative | Low | Ultra | LNRM |
| 130 | Positive | New patient | 1+ | Low | High | Ultra | LNRM |
| 131 | Negative | New patient | 2+ | Medium | High | Ultra | LNRM |
| 133 | Negative | New patient | 3+ | Very low | High | Ultra | LNRM |
| 135 | Unknown | New patient | 2+ | Negative | High | Ultra | LNRM |
| 137 | Unknown | New patient | 1+ | Negative | Medium | Ultra | LNRM |
| 142 | Negative | New patient | 3+ | Very low | High | Ultra | LNRM |
| **147** | Positive | New patient | Negative | Very low | Low | Ultra | LNRM |
| 148 | Negative | New patient | Scanty | Very low | Medium | Ultra | LNRM |

AFB = acid fast bacilli; ITM = Institute of Tropical Medicine, Antwerp, Belgium; LNRM = Laboratoire National de Référence de Mycobactériologie, Conakry, Guinée
